# Knowledge and Practice of Malaria Prevention Methods Among Residents of Arba Minch Town and Arba Minch Zuria District, Southern Ethiopia

**DOI:** 10.4314/ejhs.v20i3.69448

**Published:** 2010-11

**Authors:** Ayalew Astatkie

**Affiliations:** 1 Department of Public and Environmental Health, College of Medicine and Health Sciences, Hawassa University

**Keywords:** knowledge, practice, malaria, prevention methods, Arba Minch

## Abstract

**Background:**

To date, there is no effective vaccine or no effective drug for mass chemoprophylaxis against malaria. Thus, proper know-how and use of prevention methods is crucial. This study aims to assess the knowledge and practice of malaria prevention methods among the residents of Arba Minch area, Southern Ethiopia.

**Methods:**

A community-based cross-sectional study that utilized a two-stage sampling was conducted from January 22 to February 1, 2007 on a sample of 454 household heads or their deputies. SPSS 16 for windows was used for data analysis. Chi-square and Fisher's exact probability tests were used to assess the association of selected variables with place of residence.

**Results:**

Majority (86.8%) of the respondents mentioned fever as a symptom of malaria, and 98.2% of the respondents reported mosquito bite as the cause of malaria. Three hundred and eighty four (84.6%) of the respondents mentioned mosquito nets as protective measures against mosquito bites. The protective measure mostly used by the respondents or other household members in the last 12 months was mosquito net (73.3%) followed by aerosol insecticide (13%) with the former being used more in rural areas and the latter in urban areas.

**Conclusion:**

The study subjects' awareness regarding the cause, symptoms and preventive measures of malaria was high. Use of mosquito net as protective measure against mosquito bites in the last 12 months was high. However, use of other preventive measures was low. Behavioral change communication is required to increase the use of other preventive measures along with mosquito nets.

## Introduction

Malaria has continued to be a major threat to the world's community posing its huge toll of morbidity and mortality in Sub-Saharan Africa (SSA) (1–5). And of the disease's burden in SSA, the lion's share is levied on children (1). Pregnant mothers are also put at a more risk due to altered level of immunity (5). The disease is also incriminated as a cause for reduction in economic growth (2).

Likewise, in Ethiopia, the disease is a major public health problem (6–8). Three- quarters of the land mass (altitude < 2000 meters) is suitable for malaria (9), and two-third of the population lives in malarious areas (3, 7–9). Transmission is seasonal and unstable (6, 9), and case fatality is 17–35% (9). Due to lack of communal immunity, the population is at risk of repeated epidemics (6).

To make things worse, to date, there is no vaccine or no safe, effective and affordable drug for mass chemoprophylaxis against malaria (7, 10). Yet, the disease can effectively be prevented using the available preventive interventions (11). The recommended preventive interventions are use of insecticide treated mosquito nets (ITNs) and, indoor residual house spraying and other preventive interventions where appropriate and effective (1). The term “*other preventive interventions*” in the preceding sentence is broad and embodies several possible interventions including environmental management. Thus, people's proper knowledge and practice of the available preventive methods is essential. Because practice is the application of knowledge (12), people should first have a good knowledge base of the available methods. Then, they will make use of the methods based on what they know.

So far, a study assessing utilization of ITNs in Arba Minch Town and its surrounding malarious rural villages had been published (13). However, knowledge on malaria and its prevention measures and practice of preventive measures other than ITNs were not reported in that article. This article is thus designed to survey the knowledge and practice of the population in the stated study locality on malaria and its preventive actions.

## Materials and Methods

The materials and methods used to carry out the study also described elsewhere (13) are presented below.

The study used a community-based cross-sectional study design to assess the knowledge and practice of the residents in Arba Minch Town and the malarious villages of its surrounding District on malaria and its prevention methods. Utilization of insecticide treated nets (ITNs) among the households of the stated locality was also assessed as a principal objective of the study and published elsewhere (13).

This study was conducted in Arba Minch Town and Arba Minch Zuria district. The centre of the study area, Arbaminch town, is located 505 Kms south of Addis Ababa, the capital city of Ethiopia. It is found at an altitude of 1200–1300 meters above sea level with an average annual temperature of 29.7°C and rain fall of 700 mm. The rural villages of Arba Minch Zuria district are found at an altitude ranging from 1187 Kms to a peak of 2700 meters above sea level with an average annual temperature of 23.6°C and a rainfall of 950 mm. Arba Minch town is administratively divided in to four *kifle-ketemas* (sub-towns) which are further subdivided into sixteen *kebeles* all of which are malarious. Arba Minch Zuria District consists of 30 kebeles of which 11 are malarious.

The total population of the study area (Arba Minch Town and Arba Minch Zuria district) was 141, 779 during the study period. Of these, 6040 (4.26 %) were pregnant women and 25, 677 (18.11%) were children under five years of age. The total number of households was 28, 354 with an average household size of 5. The dominant income generation sources were trade and agriculture for the town and the rural population respectively. (Information regarding the study area is obtained by personal communication with relevant offices.)

The study population were household heads or their deputies (often and preferably wives) who were permanent residents in selected *kebeles* of Arba Minch Town and Arba Minch Zuria district.

The sample size was calculated using the formula for estimating a single proportion, $n=\frac{p(1-p)}{w^{2}}$. The assumptions made were: an expected proportion (ITN possession as a proxy measurement of practice of malaria prevention methods) of 11% based on the 2004 NetMark survey in Ethiopia (14), 95% confidence level and a 3% tolerable error. Accordingly, the sample size required for this study was 413 respondents. Adding 10% for non-response, the grand total sample size required was 454.

In the sampling process, *kebeles* were stratified into urban and rural places and sample size allotted proportionally. The ratio of urban-to-rural sample was nearly one-to-one. Subsequently, a two-stage sampling was accomplished. First, 8 *kebeles* (4 urban and 4 rural) were selected randomly using the lottery method from a total of 27 (16 urban and 11 rural) malarious *kebeles*. As a result, from Arba Minch town, *kebele 03, kebele 05, kebele 07* and *kebele 13* were selected. Likewise, from the malarious villages of Arba Minch Zuria District, *kebeles* named *Elgo Fango, Ganta Kanchama, Kolla Shara* and *Kolla Shelle* were selected. Next, data collectors went to the approximate centre of each selected *kebele* and spun a pen. Then the households towards which the ball point of the pen indicated were serially selected. The number of households which were selected in each *kebele* was proportional to the total number of households in the *kebele*.

Data were collected from 22^nd^ January to 1^st^ February 2007 using structured, pre-tested and interviewer-administered questionnaire. Some questions in the questionnaire were adapted from the NetMark Baseline Household Evaluation Survey Instrument (15). The questionnaire was prepared in English and then translated into Amharic for the data collection. The questionnaire included questions that address relevant socio-demographic information about the respondents, knowledge about malaria, mosquitoes and malaria prevention methods and practice of malaria prevention methods.

The interview was carried out by 8 experienced interviewers after providing a thorough training on the data collection procedure. As there were several possible alternatives for some of the questions pertaining to knowledge and practice, the interviewers first read the alternative responses to the respondents before they gave answers. In households where there were married couples, the husband or the wife (preferably the wife if both were available at the same time) was the respondent. In other circumstances (when there were no married couples), the head of the household responded for the interview. If the appropriate respondent was not available in the house during initial visit, revisits were considered to contact the appropriate person.

Data entry and analysis was performed using the Statistical Package for Social Sciences (SPSS) 16.0 for windows and the results were summarized using tables. Chi-square test and Fisher's exact probability test were carried out to assess associations between place of residence and knowledge and practice variables. The level of significance (*α*) at 0.05 was used for all tests of statistical significance.

Ethical clearance was obtained from the Research and Publication office of the University of Gondar. After getting ethical clearance, written permission was obtained from Gamogofa Zone Health Department, Arba Minch Town Administration Health Office and Arba Minch Zuria District Health Office. Verbal consent was also obtained from each individual respondent during data collection after a through explanation of the purpose of the study.

## Results

All the proposed 454 study subjects were interviewed. Urban and rural respondents constituted 222 (48.9%) and 232 (51.1%) of the study subjects respectively. One hundred and six (23.3%) of the respondents were males while the remaining 348 (76.7%) of the respondents were females. In terms of responsibility in the household, 179 (39.4%) of the respondents were heads of the household and the rest 275 (60.6%) were wives of the household head (Table 1).

**Table 1:** Socio-demographic background of the study population, Arba Minch Town and the malarious areas of Arba Minch Zuria district, Southern Ethiopia, Feb. 2007.

	Variables (n=454)	Number	Percent
Place of residence			
Arba Minch Town			
	Total	222	48.9
	*Kebele 03*	63	13.9
	*Kebele 05*	53	11.7
	*Kebele 07*	47	10.4
	*Kebele 13*	59	13
Arba Minch Zuria District			
	Total	232	51.1
	*Elgo Fango*	74	16.3
	*Ganta Kanchama*	22	4.8
	*Kolla Shara*	61	13.4
	*Kolla Shelle*	75	16.5
Sex of respondent			
	Male	106	23.3
	Female	348	76.7
Responsibility of respondent in the household			
	Head of the household	179	39.4
	Wife of head of the household	275	60.6

All the 454 (100%) respondents had information about malaria and 394 (86.8%) of the respondents mentioned fever as a symptom of malaria. Chills, headache, nausea/ vomiting, loss of appetite and body/ joint aches were pointed out by 77.3%, 64.8%, 46.7%, 40.8% and 31.5% of the respondents respectively as possible symptoms of malaria. Four hundred and forty six (98.2%) of the respondents mentioned being bitten by mosquitoes as the cause of malaria. The respondents also supposed stagnant water (34.4%), hunger (30.0%), overwork (4.6%), and drinking dirty water (3.7%) as causes of malaria. Children under five years and pregnant women were mentioned by 410 (90.3%) and 268 (59.0%) of the respondents respectively as the people most likely to be seriously affected by malaria. The Rainy Season and the time immediately following it were mentioned by 54.9% and 49.6% of the respondents respectively as the times in a year when mosquitoes bother or bite the most. Three hundred and ninety six (87.2%) of the respondents replied that mosquitoes bother or bite the most at night during sleeping (Table 2).

**Table 2:** Knowledge of respondents related to symptoms, causes and risks of malaria, Arba Minch Town and the malarious villages of Arba Minch Zuria district, Southern Ethiopia, Feb. 2007.

	Variable (n=454)	Number	Percent
Ever heard of the disease malaria			
	Yes	454	100
	No	0.00	0.00
Symptoms of malaria mentioned*			
	Fever	394	86.8
	Chills	351	77.3
	Headache	294	64.8
	Nausea/ vomiting	212	46.7
	Loss of appetite	185	40.8
	Body/ joint aches	143	31.5
	Diarrohoea	35	7.7
	Weakness	24	5.5
	Feeling bitter taste	24	5.3
	Dizziness	22	4.8
	Others	10	2.2
Cause of malaria mentioned*			
	Being bitten by mosquitoes	446	98.2
	Stagnant water	156	34.4
	Hunger	136	30.0
	Overwork	21	4.6
	Drinking dirty water	17	3.7
	Don't know	3	0.7
	Other	5	1.1
People most likely to be seriously affected by malaria*			
	Children under 5 years	410	90.3
	Pregnant women	268	59.0
	Non-pregnant women	33	7.3
	Adult men	29	6.4
	Don't know	24	5.3
Seasons in the year when mosquitoes bother or bite the most*			
	Rainy season	249	54.9
	Following a rainy season	225	49.6
	Dry season	13	2.9
	Throughout the year	3	0.7
	Don't know	2	0.4
Time in a day when mosquitoes bother or bite the most*			
	At night during sleeping	396	87.2
	At night before sleeping	86	18.9
	Other	14	3.1
	Don't know	3	0.7

* More than one response possible.

Four hundred and fifty two (99.6%) of the respondents have got information about how to avoid getting malaria in the 12 months preceding the study. The principal source of information was health personnel (346 (76.6%)) followed by radio (269 (59.5%)).

Mosquito nets (treated/ untreated/ unspecified) were mentioned by 433 (84.6%) of the respondents as protective measures against mosquitoes. Furthermore, 247 (54.4%) of the respondents pointed out keeping surroundings clean, 167 (36.8%) cited closing windows/ doors, 115 (25.3%) indicated smoking rooms with olive twig, sugarcane, etc, and 109 (24.0%) stated insecticidal aerosols as protective measures against mosquito bites.

Regarding the advantages of sleeping under mosquito net, 415 (91.4%) of the respondents mentioned that it avoids getting bitten by mosquitoes, 214 (47.1%) stated that it avoids getting malaria, and 78 (17.2%) replied that it protects other insects/ pests (e.g.: lice, bed buds, fleas, etc). Three hundred and seventy two (81.9%) of the respondents responded that mosquito nets have no disadvantage. With regard to the advantage of ITNs relative to untreated nets, 415 (96.5%) of those who have heard of ITNs reported that it kills mosquitoes, 247 (57.4%) mentioned that it repels mosquitoes away from the net, and 143 (33.3%) replied that it kills/ repels other insects/ pests (e.g.: lice, bed bugs, fleas, etc) (Table 3).

**Table 3:** Knowledge of respondents about malaria prevention methods, Arba Minch Town and the malarious villages of Arba Minch Zuria district, Southern Ethiopia, Feb. 2007.

	Variable (n=454)	Number	Percent
Heard about how to avoid getting malaria in the last 12 months			
	Yes	452	99.6
	No	2	0.4
Source of information about how to avoid getting malaria (n=452)*			
	Health personnel	346	76.6
	Radio	269	59.5
	Television	93	20.6
	Friends/ neighbours/ relatives	54	12
	News paper/ magazines/ Posters/ notice at a pharmacy	20	4.4
	Can't recall	1	0.2
Methods or products mentioned to be protective against mosquitoes*			
	Mosquito nets (treated/ untreated/ unspecified)	433	95.4
	Keeping surroundings clean	247	54.4
	Closing windows/ doors	167	36.8
	Smoking (olive twig, sugar cane, etc)	115	25.3
	Insecticidal aerosols	109	24.0
	Protective clothing	29	6.4
	Commercial mosquito repellants applied on the body	16	3.5
	Other	21	4.7
	Don't know any	2	0.4
Ever heard of a mosquito net			
	Yes	454	100
	No	0	0
Advantages of sleeping under a mosquito net*			
	Avoids getting bitten by mosquitoes	415	91.4
	Avoids getting malaria	214	47.1
	Protects other insects (e.g.: lice, bedbugs, fleas, etc)	78	17.2
	Avoids the disturbing buzzing sound of mosquitoes	16	3.5
	Other	16	3.5
	Don't know	1	0.2
Disadvantages of sleeping under a mosquito net*			
	No disadvantage	372	81.9
	It is hot sleeping under a net	29	6.4
	Other	10	2.2
	Don't know	44	9.7
Ever heard of ITNs			
	Yes	430	94.7
	No	24	5.3
Advantage of ITNs relative to untreated nets (n=430)*			
	Kills mosquitoes	415	96.5
	Repels mosquitoes away from the net	247	57.4
	Kills/ repels other insects/ pests (e.g.: lice, fleas, bed bugs, etc)	143	33.3
	It is better at preventing malaria	17	4.0
	Don't know	4	0.9
Disadvantage of sleeping under ITNs (n=430)*			
	No disadvantage	372	86.5
	Smell is bad	15	3.5
	Other	13	3.0
	Don't know	32	7.4

* More than one response possible.

Mentioning fever as a symptom of malaria, identifying mosquito bite as the cause of malaria, pointing out children under five years as being highly vulnerable to malaria, and stating mosquito nets as preventive measure against mosquito bite didn't show statistically significant difference between urban and rural residents. However, identifying pregnant women as being highly vulnerable to malaria was higher among urban residents (161 (72.5%)) compared to rural residents (107 (46.1%)) (p<0.001) (Table 4).

**Table 4:** Association of selected knowledge variables with place of residence, Arba Minch town and the malarious villages of Arba Minch Zuria district, Southern Ethiopia, Feb. 2007.

Variable		Residence		χ^2^	p-value
				
		Urban	Rural		
				
		Number (%)	Number (%)		
Mentioned fever as a symptom of malaria					
	Yes	188 (84.7)	206 (88.8)	1.670	0.196
	No	34 (15.3)	26 (11.2)		
	Total	222 (100)	232 (100)		
Mentioned mosquito bite as the cause of malaria					
	Yes	217 (97.7)	229 (98.7)	Fisher's exact test	0.495
	No	5 (2.3)	3 (1.3)		
	Total	222 (100)	232 (100)		
Children under 5 years mentioned as the people highly vulnerable to malaria					
	Yes	203 (91.4)	207 (89.2)	0.637	0.425
	No	19 (8.6)	25 (10.8)		
	Total	222 (100)	232 (100)		
Pregnant women mentioned as the people highly vulnerable to malaria					
	Yes	161 (72.5)	107 (46.1)	32.968	<0.001
	No	61 (27.5)	125 (53.9)		
	Total	222 (100)	232 (100)		
Mosquito nets mentioned as preventive measures against mosquito bite					
	Yes	211 (95.0)	222 (95.7)	0.107	0.744
	No	11 (5.0)	10 (4.3)		
	Total	222 (100)	232 (100)		

Three hundred and thirty three (73.3%) of the respondents reported that one or more of the household members have used mosquito nets (treated/ untreated/ unspecified) and 59 (13%) expressed that they have used aerosol insecticides in the 12 months preceding the study to protect against mosquito bites (Table 5).

**Table 5:** Practice of malaria prevention methods, Arba Minch Town and the malarious villages of Arba Minch Zuria district, Southern Ethiopia, Feb. 2007.

Variable (n=454)		Number	Percent
Product or method used in the last 12 months by respondent or other household members*			
	Mosquito nets (treated/ untreated/ unspecified)	333	73.3
	Aerosol insecticides	59	13.0
	Smoke	51	11.2
	Keeping surroundings clean	39	8.6
	Closing doors/ windows	16	3.5
	Other	11	2.4
	No method used	33	7.3

* More than one response possible.

Use of mosquito nets in the 12 months preceding the study was statistically significantly higher among rural residents compared to urban residents. Conversely, use of aerosol insecticides and environmental management were statistically significantly higher among urban residents compared to rural residents (Table 6).

**Table 6:** Association of selected variables related to practice of malaria prevention methods with place of residence, Arba Minch Town and the malarious villages of Arba Minch Zuria district, Southern Ethiopia, Feb. 2007.

Variable		Residence		χ^2^	p-value
				
		Urban	Rural		
				
		Number (%)	Number (%)		
Mosquito nets used in the last 12 months to prevent malaria					
	Yes	131 (59.0)	202 (87.1)	45.692	<0.001
	No	91 (41.0)	30 (12.9)		
	Total	222 (100)	232 (100)		
Aerosols used in the last 12 months					
	Yes	52 (23.4)	7(3.0)	41.78	<0.001
	No	170 (76.6)	225 (97.0)		
	Total	222 (100)	232 (100)		
Environmental management used in the last 12 months to prevent malaria					
	Yes	27 (12.2)	12 (5.2)	7.058	0.008
	No	195 (87.8)	220 (94.8)		
	Total	222 (100)	232 (100)		

## Discussion

This paper presented the findings of a study on knowledge and practice of malaria prevention methods among residents in a malaria-endemic area in southern Ethiopia. The findings of this study, thus, may not reflect the situation in non malaria-endemic areas. Besides, the responses on practice were obtained for the 12-months period preceding the study. Hence, on some of the findings related to practice, there could be a possibility of recall bias. It should also be noted that as selection of respondents started from households situated in the centres of the selected *kebeles*, respondents in the peripheral parts of the *kebeles* might be excluded. Besides, as the sampling technique used was proximity sampling, there might be clustering of respondents with similar characteristics. The findings of this study must therefore be interpreted keeping these limitations in mind.

In this study, most of the respondents correctly identified the different symptoms of malaria. Identification of *fever* as a symptom of malaria was very high (86.8%). Other studies (16, 17) have also documented similar results in this regard. Comparable to findings elsewhere (16–19), nearly all (98.2%) of the respondents correctly pointed out being bitten by mosquitoes as the cause of malaria. This figure is, however, very much higher than that reported by the NetMark survey (14) in which only 37% of the respondents reported mosquito bite as the cause of malaria. This difference could be explained by the time gap between the two studies—the NetMark survey was carried out about three years before this study. In this study, misconceptions about the cause of malaria were also noted; respondents perceived stagnant water, hunger, overwork and drinking dirty water as the causes of malaria. The fact that the respondents perceived stagnant water as the cause of malaria could be because mosquitoes breed on stagnant water and thus people living in areas where there is stagnant water may suffer more from the problem of malaria than people living in areas where there is no stagnant water. This perception may be more of useful than harmful because such people may be more willing to take environmental measures such as draining of mosquito breeding sites. Despite this fact, the misconceptions regarding the cause of malaria should be changed with appropriate behavioral change communication (BCC) because they may have an influence on the actual preventive measures against malaria. Fore example, a person who perceives hunger as the cause of malaria may think that eating to satisfaction is all that is needed to avoid malaria and may fail to take measures against mosquito bites.

Identification of children under five years of age as a group highly vulnerable to malaria was high (90.3%) but somewhat lower for pregnant women (59%). This finding is comparable to findings obtained elsewhere (14, 16). Based on these findings, the vulnerability of pregnant women seems to be underestimated. This must be changed with appropriate BCC intervention because unless the vulnerability of pregnant women is well recognized by the population, they may not be given priority to use proper preventive measures such as ITNs.

Awareness of the different methods of protecting mosquito bites, especially that of mosquito nets, in this study is high (84.6%) which is almost in agreement with the result found in Kafta-Humera, Tigray region, by Haileselassie B (16). Mosquito nets were recognized by the participants of this study for avoiding getting bitten by mosquitoes, avoiding getting malaria and protecting other insects/ pests (e.g.: lice, fleas, bed bugs, etc). The perceived advantage that mosquito nets protect against other insects may give the nets an added value and thereby enhance their acceptability and importance.

It is also encouraging that most (73.3%) of the households used mosquito nets in the last 12 months to protect one or more of the household members from mosquito bites. The second mostly used protective measures were insecticidal aerosols (13%). Implicit within this fact is that mosquito nets are used and probably accepted much more than other alternative measures in the study area. Though environmental management (keeping surroundings clean) was mentioned by 54.4% of the respondents as one of the protective measures against mosquitoes, this intervention is reportedly used only by 8.6% of the households. The people must, therefore, be encouraged (with BCC interventions) to implement this very important measure together with other measures such as ITNs to score a maximal malaria reduction impact.

In conclusion, based on the findings of this study, it is evident that the level of awareness of the respondents regarding the cause, symptoms and preventive measures of malaria was high. Yet, misconceptions about the cause of malaria were not uncommon. Though the vulnerability of children under five years to malaria was highly recognized, the vulnerability of pregnant women to the disease was underestimated. The advantages of sleeping under mosquito nets were also well recognized. Use of mosquito net as protective measure against mosquito bites in the 12 months preceding the study was high. However, use of other preventive measures was low.

Appropriate behavioral change communication interventions should be implemented to clear the misconceptions related to the cause of malaria and to raise the awareness on the vulnerability of pregnant women. Interventions which are well recognized but less used by the people such as environmental sanitation must be given attention and their use concomitantly with mosquito nets be encouraged by health extension workers and other health educators.
